# Are TADs supercoiled?

**DOI:** 10.1093/nar/gky1091

**Published:** 2018-11-05

**Authors:** Dusan Racko, Fabrizio Benedetti, Julien Dorier, Andrzej Stasiak

**Affiliations:** 1 Center for Integrative Genomics, University of Lausanne, 1015 Lausanne, Switzerland; 2 SIB Swiss Institute of Bioinformatics, 1015 Lausanne, Switzerland; 3 Polymer Institute of the Slovak Academy of Sciences, 842 36 Bratislava, Slovakia; 4 Vital-IT, SIB Swiss Institute of Bioinformatics, 1015 Lausanne, Switzerland

## Abstract

Topologically associating domains (TADs) are megabase-sized building blocks of interphase chromosomes in higher eukaryotes. TADs are chromosomal regions with increased frequency of internal interactions. On average a pair of loci separated by a given genomic distance contact each other 2–3 times more frequently when they are in the same TAD as compared to a pair of loci located in two neighbouring TADs. TADs are also functional blocks of chromosomes as enhancers and their cognate promoters are normally located in the same TAD, even if their genomic distance from each other can be as large as a megabase. The internal structure of TADs, causing their increased frequency of internal interactions, is not established yet. We survey here experimental studies investigating presence of supercoiling in interphase chromosomes. We also review numerical simulation studies testing whether transcription-induced supercoiling of chromatin fibres can explain how TADs are formed and how they can assure very efficient interactions between enhancers and their cognate promoters located in the same TAD.

## INTRODUCTION

### From topological domains to TADs

Many of us are familiar with the famous dictum attributed to Jacques Monod ‘Anything found to be true of *Escherichia coli* must also be true of elephants’ (1). That dictum nicely grasps the essence of the unifying principle of biology that applies to such fundamentals of life as DNA structure, genetic code, gene regulation, mechanisms of DNA replication etc. Of course, the details of a given process, like of DNA recombination, for example, can differ between *E. coli* and elephant, i.e. between prokaryotes and higher eukaryotes, but such general features like action of recombinase proteins that stretch and unwind the DNA during the homologous paring process are universal (2).

For >50 years now, it has been known that bacterial chromosomes with their several MB long circular DNA molecules are organized into topological domains, where sequential, 5–50 kb large portions of DNA form supercoiled loops (3–5). We progressively started to appreciate the significance of topological domains with respect to such aspects as giving bacterial chromosomes an organized structure (5) or providing the possibility of having different supercoiling level in different topological domains, which in turn permits a differential control of gene expression (6). Knowing the importance of topological domains for gene regulation in bacteria, it is natural to consider whether organization of chromosomes into supercoiled loops, that can vary their supercoiling level, belongs to fundamental biological principles that are maintained during evolution from prokaryotes to higher eukaryotes.

At least since 1977, when the well-known now electron microscopy pictures of histone-depleted metaphase chromosomes were published by Paulson and Laemmli (7), it has been considered as demonstrated that metaphase chromosomes of higher eukaryotes are composed of ca 100 kb long loops. The chromosome loops were also proposed to organize interphase chromosomes (8), where at least part of these loops involved matrix attachment regions interacting with nuclear lamin (9) and type II DNA topoisomerases bound to chromatin near loop attachment sites (10). These loops were frequently called topological domains (11), without directly implying though that they are supercoiled, as that is the case of bacterial topological domains. However, it was also shown that many of detected chromatin loops resulted from non-specific chromatin aggregation (12) and there were no chromatin loops revealed by cryo-electron microscopy studies of chromosomes (13). The lack of consensus in the field contributed to the fact that the concept of organization of interphase chromosomes into specific loops gave stage to other developments in chromosome research that in the last two decades were connected to progress in genomics methods. The view that dominated the field and textbooks at the turn of 21^st^ century was that during interphase stage of the cell cycle, chromatin fibres just behave like very long linear polymers that, due to their large size, require very long time to equilibrate (14–16). Only quite recently, though, thanks to very rapid development of high-resolution 3C (Chromosome Conformation Capture) methods, almost everybody in chromosome structure field started to think and talk about topological domains in interphase chromosomes of higher eukaryotes. In 2012, three seminal papers using advanced 3C methods revealed that human, mice or drosophila chromosomes are composed of ca 1MB large, self-interacting regions that follow each other along individual chromosomes (17–19). On average, for the same genomic distance, two chromosomal loci contained in the same self-interacting domain contact each other 2–3 times more frequently than two loci located in two neighbouring domains. The title of one of those seminal papers even announced the identification of topological domains in mammalian genomes (17). The term ‘topological domains’ implicitly suggested that the structure of self-interacting domains in eukaryotic chromosomes is likely to be similar to the structure of topological domains in bacterial chromosomes. However, the model of self-interacting domains proposed in that seminal paper of Dixon *et al*. was very different from the structure of supercoiled topological domains in bacterial chromosomes. Individual topological domains in mammalian chromosomes were schematically presented as quasi-spherical globules (17). Dixon *et al*. did not discuss though what underlying physical and biological mechanisms could be responsible for the formation of proposed chromatin globules with increased frequency of internal contacts. Supercoiling was not considered as a possible mechanism of compacting individual self-interacting domains (17). In another paper published in the same issue of Nature, Nora *et al*. mapped positions of self-interacting chromatin domains in a specific region of mice interphase X chromosome (18). Nora *et al*. named these self-interacting chromatin domains as topologically associating domains (TADs). Since then, the acronym TAD is very widely used and almost needs no explanation in today's papers on chromosome structure.

### Models of TADs

Probably the first model of TADs formation was proposed by Barbieri *et al*. (20). In their model, chromatin portions constituting different TADs were assumed to have different epigenetic marks, which in turn bind mark-specific polyvalent binders. Polyvalent binders compactify then individual TADs, which in turn increases the frequency of intra-TAD contacts. That model of TADs formation requires that neighbouring TADs should have different epigenetic marks, as otherwise such neighbouring TADs would fuse. However, at least in mammalian cells, large chromatin portions with the same epigenetic state (transcriptionally active, or inactive, for example) are still composed of individual TADs (17). Therefore, some other mechanism should be responsible for TADs formation in chromosomal portions with the same epigenetic state.

**Figure 1.**
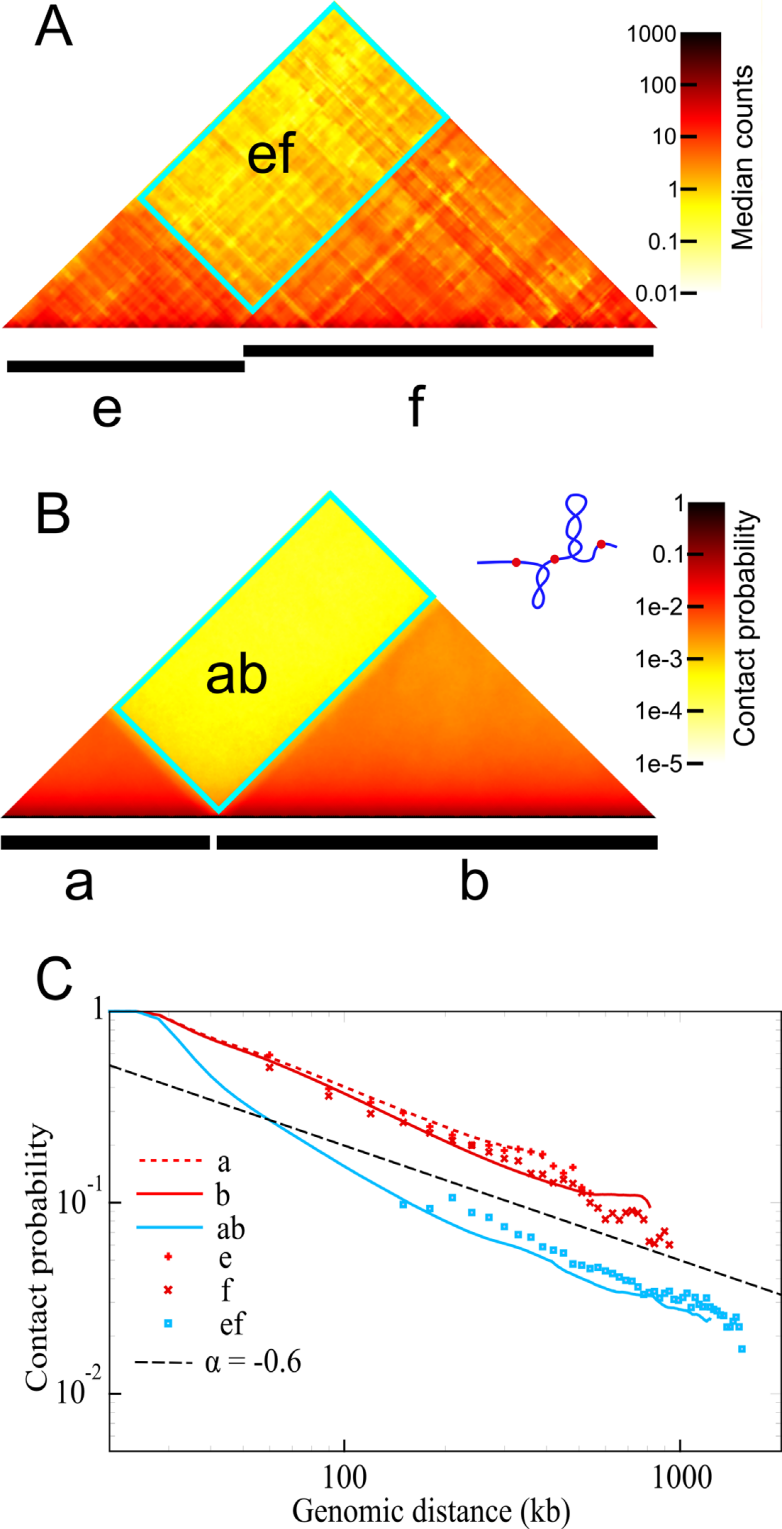
Models of supercoiled TADs show consistency with experimental data. (**A**) Experimental contact map of a short chromosome region with two TADs. That map was generated using the deposited data (18) and shows two TADs that were indicated with letters E and F in the contact map shown in Figure 1c of Nora *et al*. paper (18). (**B**) Contact maps obtained in a simulation of two neighbouring chromatin loops that were modelled as supercoiled elastic polymers with properties approximating physical behaviour of chromatin fibres with 400 and 800 kb, respectively. In this simulation the supercoiling density was set to two negative superturns for every 100 kb. (**C**) Comparisons between the experimental and simulated contact frequency profiles. Experimental data for the genomic region shown in (A) are from Nora *et al*. (18). The experimental results are shown as individual data points, whereas simulated results form continuous profiles. The straight dashed line shows the slope, which would correspond to the rate of contact decrease characterized by the α exponent of –0.6. Intra-TAD contacts determined experimentally or by simulations are indicated with one letter description of the corresponding TADs. Two letter descriptions indicate corresponding inter-TAD contacts. Red and blue colour relate to intra- and inter-TAD contacts, respectively. Notice that simulations give directly the contact probability whereas experimental data give only relative contact probabilities. Therefore, on log/log plots, the experimental data points can be all together shifted vertically by the same rescaling factor during the fitting procedure. Figure 1 is adapted from (21).

Modelling studies inspired by bacterial topological domains tested whether supercoiling of chromatin fibres can reproduce several known features of eukaryotic chromosomes (21). Models where individual TADs formed a few plectonemic superturns reproduced qualitatively the experimental contact maps (Figure 1). This included ca. 2–3-fold increase of intra-TAD contacts as compared to inter-TADs contacts, as well as the scaling relation between the probability of contacts and the genomic distance between interacting loci, which for genomic distances of the order of TAD sizes was characterized by the scaling exponent α of –0.6 (18).

Although models assuming supercoiling of chromatin fibres forming individual TADs were able to qualitatively reproduce the experimental data (21), the question remains whether TADs are indeed supercoiled and what are the mechanisms of supercoiling generation in eukaryotic chromosomes. Eukaryotes, in contrast to bacteria, do not have gyrase that is a specialized DNA topoisomerase that actively supercoils the DNA using the energy gained from ATP hydrolysis. However, transcription is known to be a potent generator of supercoiling both in prokaryotes and eukaryotes. Since late 1980ties it is known that transcribing RNA polymerases by binding to various nuclear components, including other RNA polymerases are prevented from encircling the transcribed template (22,23). As a consequence, transcribed DNA and thus transcribed chromatin fibres are forced to undergo axial rotation (24–28). The axial rotation of chromatin fibres is generated at sites where helical DNA is actually passing through RNA polymerase. The transmission of rotational motion to flanking portions of chromatin fibres is however slow in viscous nuclear environment that limits diffusion of torsional stress along chromatin fibres (29) and even a friction between DNA and interior of type I topoisomerases limits swivelling motion needed for the relaxation of the torsional stress (30). As a consequence, negative supercoiling accumulates behind transcribing polymerases, whereas ahead of transcribing polymerases one observes formation of positive supercoiling (24–27). Cellular DNA topoisomerases can relax both positive and negative supercoiling. However, the efficiency of relaxation of positive and negative supercoiling are not the same (31,32). Positive supercoiling is relaxed more rapidly as its accumulation is more harmful for the cells by its inhibitive effect on the process of DNA replication and transcription (32). Therefore, ongoing transcription and action of cellular topoisomerases can maintain complex dynamic equilibrium of DNA supercoiling where different portions of chromosomes can have different levels of DNA supercoiling. Numerous studies over the last 30 years provided fragmentary view on supercoiling of specific portions of the genome (33–35). Only during the last decade, with the development of modern genomic approaches, we gained insights into the global supercoiling landscape of eukaryotic chromosomes (31,36–38). A convenient method to study DNA supercoiling is based on the quantification of the level of binding of intercalating agents such as psoralen to DNA (31). Binding of intercalating drugs decreases torsional stress in negatively supercoiled DNA, whereas it increases the torsional stress in covalently closed non-supercoiled DNA or in positively supercoiled DNA. Therefore, when psoralen is added at low concentration, the probability of its binding and crosslink formation increases with the magnitude of negative supercoiling of a given topological domain, where it can be detected and quantified upon UV crosslinking (31). Differential levels of psoralen binding to various genomic regions can be detected using such genomic approaches as high-resolution microarrays (36). Using this approach, Naughton *et al*. have provided the evidence that human interphase chromosomes are divided into domains with various levels of supercoiling (36). Actively transcribed regions showed high affinity to psoralen leading to a conclusion that they are negatively supercoiled. The transcriptionally inactive regions showed diminished affinity to psoralen, which is consistent with the idea that these regions are non-supercoiled. Diminished level of psoralen binding to transcriptionally inactive chromatin regions could be also caused by positive supercoiling resulting from formation of additional nucleosomes, as each formed nucleosome introduces one positive supercoil (39). Transcriptionally inactive regions may also be bound by heterochromatin binding proteins that make DNA less accessible to psoralen.

Both, transcription and topoisomerase activities were shown to be required to maintain the steady state dynamic profile of supercoiling with chromosomal portions showing various levels of psoralen binding. The fact that neighbouring chromatin domains frequently show very different affinity to psoralen indicated that these domains form chromatin loops with different levels of supercoiling and these loops are separated by borders that do not permit the supercoiling to diffuse between neighbouring loops (36). In addition, Naughton *et al*., reported that the borders between chromatin regions with different levels of DNA supercoiling frequently corresponded to inter TADs borders detected by 3C methods (36). However, on average, chromatin domains with a quasi-constant level of DNA supercoiling had the median size of ca 100 kb, which is ca 10 times smaller than the average size of TADs determined in the early studies (17) but is a typical size of smaller loops within TADs known as sub-TADs (40). It would be very informative to compare high-resolution Hi-C-determined borders of TADs and sub-TADS with the borders of supercoiling domains determined by Naughton *et al*. (36).

Using a similar genomic approach based on preferential binding of psoralen to negatively supercoiled DNA, Kouzine *et al*. mapped more precisely where transcription-induced supercoiling localizes (37). Meta-analysis of all regions surrounding transcription start sites (TSS) of active genes in human cells indicated that transcription-generated negative supercoiling was strongest near TSSs (37). Traces of transcription-induced positive supercoiling were detected ahead of transcribing polymerases. However, the magnitude of positive supercoiling was much smaller than the magnitude of negative supercoiling detected near TSS (37). This later result indicates that positive supercoiling is relaxed much quicker by cellular topoisomerases than negative supercoiling. Similar conclusion was also reached in earlier studies of transcription-induced supercoiling in yeast (32). Also, recent studies of transcription in human cells revealed that human topoisomerase 1, which is associated with the transcribing RNA polymerases, preferentially relaxes supercoiling in the gene's body but not at promoters (41). According to the twin-domain model, each transcribing RNA polymerase is a node between positive and negative supercoils. Therefore, between pairs of elongating polymerases, positive and negative supercoils cancel each other out. This suggests that Top1 preferentially relaxes the positive supercoiling generated by the first RNA polymerase, which is likely to be achieved by the placement of Top1 in front of the advancing RNA polymerase. As transcribing RNA polymerase is a topological barrier that blocks the diffusion of supercoiling (22), only placement of Top1 in front of RNA polymerase can relax positive supercoiling that opposes the transition to the elongation phase of transcription (41). Preferential relaxation of transcription-induced positive supercoiling, as compared to relaxation of negative supercoiling, implies that transcribed regions are actively maintained in the state of negative supercoiling. These results thus agree with the generalized presence of negative supercoiling in gene-rich regions of human chromosomes (36). Although part of Kouzine *et al*. results can be interpreted as showing that regions with negative supercoiling persist only within relatively short chromatin portions behind transcribing polymerases, this interpretation depends on somewhat arbitrary setting of the base line, i.e. the level of psoralen binding above which one considers chromatin as negatively supercoiled (37). Therefore, a safer interpretation of Kouzine *et al*. studies is that negative supercoiling is strongest just behind transcribing polymerases and then decays but can extend much further, as in fact shown in their Figure 3b (37).

### Facilitation of local separation of DNA strands by negative supercoiling

Early studies addressing the role of negative supercoiling in bacteria revealed that negative supercoiling, which is known to facilitate local strand separation, stimulates the process of transcription (42). The facilitation of local strand separation by negative supercoiling should also apply to eukaryotic transcription and numerous experiments showed that this is indeed the case (43,44). Thus negative supercoiling in TADs containing active genes is expected to create favourable environment for further gene expression (45). On the other hand, positive supercoiling or just the absence of negative supercoiling make the strand separation more difficult, which in turn would inhibit initiation of transcription. In this respect the observation of Naughton *et al*. (36) that negatively supercoiled chromosomal domains contain active genes, whereas positively or less negatively supercoiled domains group inactive genes suggests that supercoiling by its direct effect on the stability of DNA may be used to regulate gene expression in eukaryotic cells.

### Supercoiling stimulates legitimate enhancer-promoter contacts

As already discussed (see Figure 1), supercoiling of individual TADs would compactify them and therefore would increase the frequency of internal contacts. Therefore, supercoiling of individual TADs would provide a physical explanation of why, on average, a pair of loci located in the same TAD contact each other more frequently than a pair of loci separated by the same genomic distance but located in neighbouring TADs. This increase of the average intra-TAD contacts is of course important for enhancer-promoter contacts and explains in part the fact that enhancers and their cognate promoters are located in the same TAD (46). However, knowing how critical is the proper enhancer-promoter interaction for cellular differentiation and ontogenesis of complex organisms, such as humans (46), one realizes that the predicted 2–3-fold difference in the contact probability between legitimate (cognate) and illegitimate (non-cognate) enhancer-promoter pairs is unlikely to be sufficient to guarantee a robust, error-free development of multicellular organisms. The 2–3-fold preference for cognate contact would result in 1 non-cognate contact for two or three cognate contacts, which would result in ectopic transcription of many genes and frequent lack of transcription of genes that should be active in a particular setting. Let us therefore reanalyse what we know about the effect of supercoiling on the contacts of genetic loci located in the same supercoiled loop.

Vologodskii *et al*., in their early simulation studies of supercoiled DNA molecules, arrived to the conclusion that supercoiling dramatically increases the frequency of intramolecular contacts between DNA sites that are far from each other along the contour of the same supercoiled DNA molecule (47). This supercoiling-induced increase of DNA–DNA contact probability was estimated to be of about two orders of magnitude as compared to non-supercoiled DNA molecules (47). These 1992 results are widely quoted and it is now a very popular notion that supercoiling dramatically increases frequency of contacts between sites located in the same supercoiled DNA molecule. However, if supercoiling were indeed increasing the frequency of intramolecular contacts by two orders of magnitude it would speak against the proposal that TADs are constituted by supercoiled loops. The frequencies of intra- and inter-TAD contacts between neighbouring TADs should then also differ by two orders of magnitude, whereas in reality there is only a 2–3 fold enhancement of intra TAD contacts as detected by 3C methods. Only in 2001, new simulation studies by Vologodskii *et al*. (48) brought a new important aspect into light. When they correctly took into account that under physiological conditions the electrostatic repulsion between DNA segments is nearly completely screened (49), the stimulation of contacts by supercoiling was greatly diminished (48). In their 1992 simulation studies Vologodskii *et al*. considered the effect of supercoiling on DNA maintained under low salt conditions i.e. conditions of strong electrostatic repulsion between DNA segments (39,49). Under these conditions supercoiling acts against electrostatic repulsion and stimulates DNA-DNA contacts that only occur very rarely in non-supercoiled DNA. Therefore, the two orders of magnitude increase of intra-molecular contacts by DNA supercoiling only applies to physiologically irrelevant regime where DNA segments strongly repulse each other. Under physiological conditions electrostatic charges of DNA and chromatin are practically neutralized which makes that DNA-DNA contacts are frequent also without supercoiling and therefore the expected stimulation of intra-TAD contacts by supercoiling becomes very modest (21).

**Figure 2.**
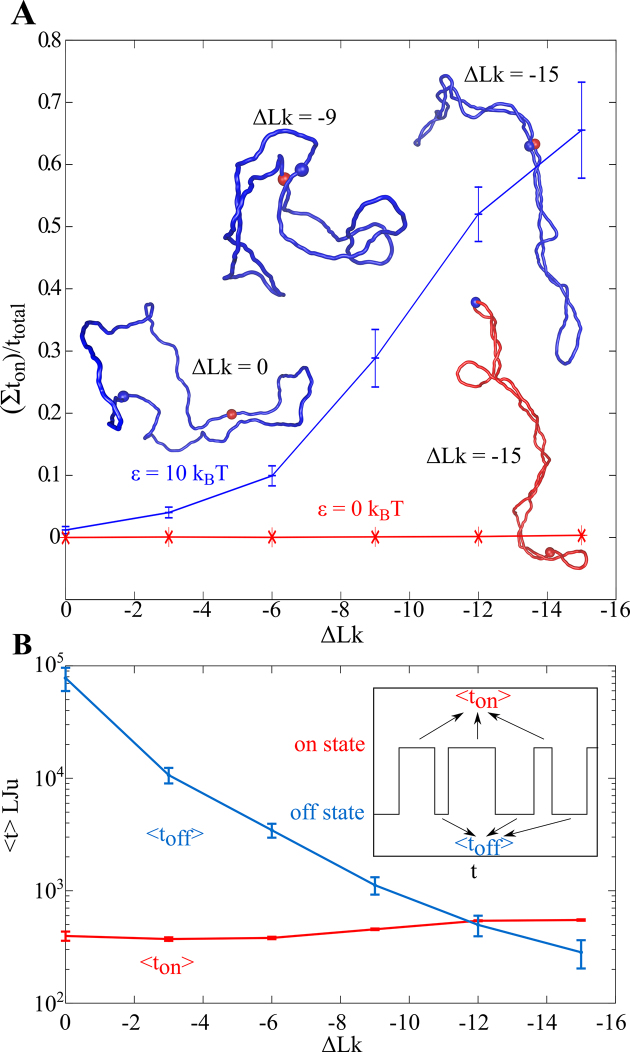
Supercoiling stimulates intramolecular interactions involving sites with mutual affinity. (**A**) Supercoiling increases the fraction of time during which a pair of sites with mutual affinity stays together, whereas interactions of sites with no mutual affinity is hardly affected by supercoiling. Simulations were set to mimic physical behaviour of 3 kb long circular DNA molecules, however qualitatively similar results are also expected for much larger, closed chromatin loops. The sites whose mutual contacts were followed, were placed 180° apart on the circular map of the modelled DNA molecules. The strength of the short-range interaction between the two sites with mutual affinity was set to 10 *k*_B_*T* (ca. 6 kcal/mol), which is in the intermediate range of strengths of protein-DNA interactions (89). The blue profile shows how the fraction of time during which two sites with mutual affinities stay together increases with increasing supercoiling. Without supercoiling that fraction of time is close to 0 since two sites with the affinity of 10 *k*_B_*T* easily dissociate due to thermal fluctuations and then in non-supercoiled DNA molecules it takes very long time till they form a new contact. The red profile shows the fraction of time during which the two sites with no mutual affinity stay together as supercoiling increases. Simulation snapshots show molecules with two sites with mutual affinity (blue) or with no mutual affinity (red). The corresponding supercoiling level is indicated. The ΔLk = –15 indicates that the linking number of the modelled DNA molecule is diminished by 15 turns as compared to torsionally relaxed DNA molecules of the same size. 3 kb long DNA molecules with ΔLk = –15 have their density of supercoiling corresponding to this of deproteinized plasmid DNA molecules isolated from healthy bacterial cells. (**B**) Supercoiling diminishes the average duration of the OFF state involving two sites with mutual affinities. The inset shows the telegraphic profile registering duration of ON and OFF states. The ON state is characterized by the distance between centres of beads representing enhancer and promoter being smaller than 6 nm. Otherwise the two beads are in the OFF state. Notice that increasing supercoiling decreases the duration of the average OFF state by over two orders of magnitude (blue profile). Figure 2 is adapted from (51).

However, this modest, 2–3-fold stimulation of contacts brings us back to the initial problem that such a low stimulation of intra TAD contacts may not be sufficient to ensure that enhancers act only on their cognate promoters located in the same TAD and are not involved in ectopic activation of genes in the neighbouring TADs. There is, however, a possible solution to this problem. In previous simulation studies investigating the frequency of contacts in supercoiled DNA molecules or chromatin fibres the authors investigated only the contacts between generic portions of modelled DNA or chromatin fibres, where the contacting portions had no mutual affinities (21,47,48). Enhancers and promoters, though, show mutual affinity mediated by specific transcription factors such as mediator or YY1, for example (50). Thus, the relevant question is how the interactions between sites that show mutual affinity are stimulated by supercoiling under ionic conditions corresponding to physiological conditions where electrostatic repulsion between DNA segments or chromatin fibres is neutralized. To answer this question Benedetti *et al*. proceeded with Brownian dynamics simulations of supercoiled DNA molecules that either were composed only of generic beads that were not attracting each other or contained in addition two distally located beads that were showing a short-range attraction to each other (51). This mutual attraction had the strength and other characteristics mimicking protein-mediated DNA–DNA interactions. The simulations revealed that in modelled DNA molecules containing only generic sites with no mutual affinity to each other the fraction of time during which a pair of pre-assigned distally located sites stayed in contact with each other was practically not increased by supercoiling (see Figure 2A). However, simulated DNA molecules containing two sites with mutual affinity reacted to increasing supercoiling by several dozen-fold increase of the fraction of time these sites with mutual affinity remained in a contact (Figure 2A). To understand better why supercoiling stimulate so strongly interactions of sites with mutual affinities Benedetti *et al*. analysed the **on** and **off** times of modelled enhancer-promoter interactions. Interestingly, the **on** times of enhancer-promoter interaction i.e. the average duration of the bound state before it undergoes thermally induced dissociation, was practically the same for supercoiled and non-supercoiled systems. However, the **off** time i.e. the average time separating the moments of dissociation from the moments of re-binding dramatically decreased as supercoiling was introduced. Shortening of the average **off** time by supercoiling indicates that after enhancer-promoter dissociation the two partners were kept close to each other. This is the consequence of the fact that supercoiling naturally keeps interwound segments in close proximity to each other and a complex and slow slithering motion is needed to move formerly interacting sites further from each other. Therefore, in supercoiled systems there is a high chance that after thermally induced dissociation of enhancer-promoter complex this complex can reform again. In case of non-supercoiled systems or in case of ectopic contacts between enhancer located in two different supercoiled TADs after thermal dissociation of such an enhancer-promoter interaction the two partners can quickly diffuse from each other, which limits their chances of quick rebinding.

**Figure 3.**
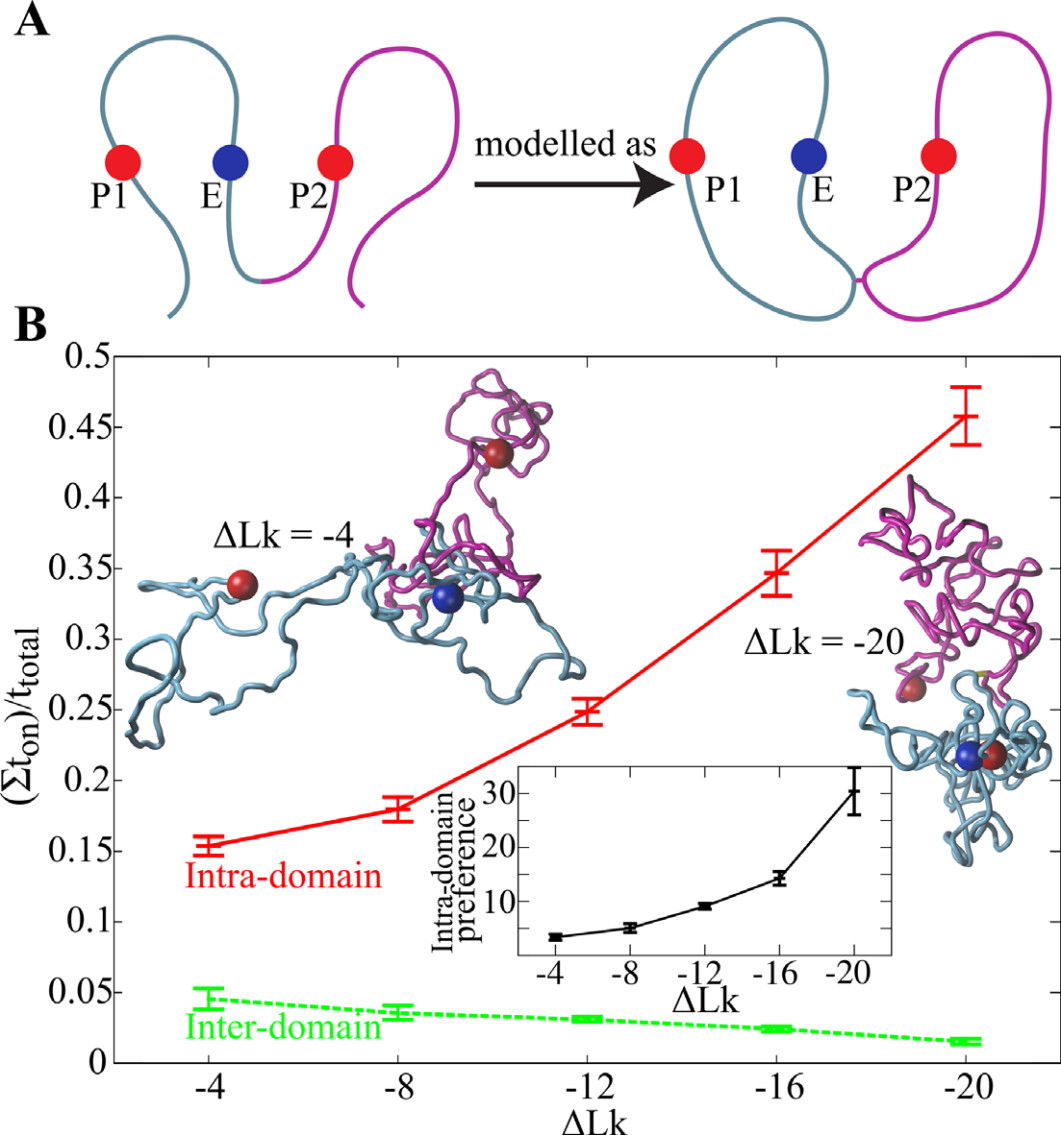
Supercoiling of TADs assures enhancer promoter fidelity. (**A**) Schematic presentation of the modelled system representing two neighbouring TADs each with the characteristics of 800 kb long chromatin loops. To be able to introduce supercoiling the two neighbouring TADs were modelled as two circular chromatin fibres connected with a linker. Bead representing enhancer, (indicated with E) shows the same strength of short-range affinity (8 *k*_B_*T*) to beads representing two promoters, (P1 and P2). The genomic distance i.e. the length of polymer chains separating E from P1 or P2, was set to be the same. (**B**) Supercoiling increases the intra-TAD preference of enhancers. The fraction of time during which enhancer (E) interacts with promoter located in the same TAD (P1) increases with increasing magnitude of supercoiling, whereas opposite effect is observed for contact between neighbouring TADs. The negative correlation between these two interactions is not set by default, as enhancer bead (E) can bind both promoter beads P1 and P2 at the same time. Representative snapshots from simulations show that for weakly supercoiled systems (ΔLk = –4) enhancer bead (E) usually does not bind any of the beads representing promoters P1 and P2. However, as the magnitude of supercoiling increases, intra-TAD interactions involving beads E and P1 are frequently observed. The inset shows how the intra-TAD preference of enhancer interaction increases with supercoiling. Figure 3 is adapted from (51).

Benedetti *et al*. simulated also the situation where an enhancer had the choice to interact with the promoter located in the same TAD or in the neighbouring TAD (see Figure 3). When both TADs were not supercoiled, the fraction of time the enhancer spent in intradomain (legitimate) interactions was ca 3 times larger than the fraction of time spent in interdomain (illegitimate) interactions. However, when both TADs were supercoiled, the intradomain (legitimate) interactions were ca. 30-fold favoured over interdomain (illegitimate) interactions. Therefore, in a biological setting where enhancers show affinity to promoters and where electrostatic repulsion between chromatin regions is largely screened, the supercoiling of chromosomal loops forming TADs can guarantee high fidelity of enhancer promoter interactions even if on average the contacts between generic sites located in the same TAD are only 2–3 times more frequent than the contacts between generic sites located in neighbouring TADs.

### Why is it advantageous to maintain large genomic distances between enhancers and their cognate promoters?

Promoters of housekeeping genes, which are constitutively expressed in every tissue, do not require activating contacts with distally located enhancers but just need interaction with their proximal regulatory elements. Due to their mutual proximity promoters and regulatory elements of housekeeping genes can find each other very efficiently. Promoters of developmentally regulated genes, however, whose expression is induced only in certain tissues or only during certain periods of development, require for their activation an interaction with distally located enhancers (52,53). Enhancers and their partner promoters are almost always located in the same TAD (46), they are frequently separated by large genomic distances which may exceed 1 MB but on average are of ∼200 kB (54). It is relatively easy to understand that regulation of genes that should be only active in certain tissues or during certain period of development may be quite complex and it is very important that these genes will not be expressed at any other time than needed. Expression of developmentally regulated genes requires co-presence of specific transcription factors where some of them bind to enhancer and others bind to promoters. Only when the entire set of transcription factors specific for a given developmental stage or tissue is present, a given set of developmentally regulated genes can be activated by interactions involving direct contact between cognate enhancers and promoters (55). However, it is difficult to understand why it is advantageous to have enhancers located up to a megabase apart from their cognate promoters. To answer this question Benedetti *et al*., performed simulations of TADs in which the effect of supercoiling was investigated on enhancer-promoter pairs placed at different genomic distances from each other (51). They have observed that when the interacting sites with mutual affinities were placed at small genomic distances from each other they very efficiently interacted with each other irrespectively of whether modelled TADs were supercoiled or not (51). However, when the interacting sites with mutual affinities were placed at large genomic distances from each other the supercoiling was needed to observe their efficient binding to each other (51). This result suggests that during evolution large genomic distances between enhancers and promoters were favoured, as only in this case the interaction between enhancers and promoters can be regulated by varying the level of supercoiling. Only distal location of enhancers with respect to promoters provides the possibility of this additional level of positive and negative control of developmentally regulated genes. Therefore, the fact that enhancers are usually located at large genomic distance from their cognate promoters supports the notion that supercoiling of chromatin can be used to regulate gene expression of genes in higher eukaryotes. This would be then similar to the role of supercoiling in gene regulation in bacteria where entire portions of chromosome can be transcriptionally activated or deactivated by changes of supercoiling level (56–58) or where transcriptional bursting depends on gyrase activity (59).

### Transcription-induced supercoiling and chromatin loop extrusion

While all TADs can be recognized on Hi-C contact maps as ‘triangles’ delineating the regions with increased frequency of internal contacts, in approximately 50% of cases the formed triangles have very strong apexes. TADs with such contact patterns form chromatin loops with their border elements contacting each other (60). ChIP-Seq studies revealed that contacting chromatin regions at the base of formed loops are bound by CTCF protein and cohesin (60). Interestingly, loop forming TADs are bordered specifically by CTCF binding sites in convergent orientation (60,61). A question of how long chromatin loops can be spanned only by contacts between CTCF proteins in convergent orientation have led to a model of chromatin loop extrusion (61–63). According to that model, individual or double rings of cohesin assembly on chromatin in such a way that a small loop is formed. That loop was proposed to grow then as cohesin rings slide along the embraced chromatin fibres (61–63). The movement of cohesin rings was proposed to stop after cohesin binds to C-terminal part of CTCF proteins and only convergent orientation of CTCF binding sites could expose C-terminal part of both CTCF proteins for stabilizing contacts with approaching cohesin rings (64). Numerical simulations testing such a mechanism closely reproduced experimental contact maps (61,62). However, until now it is not known what can drive chromatin loop extrusion in interphase chromosomes. Numerous experimental tests did not detect any DNA or chromatin translocation activity of cohesin. DNA translocation activity has been observed though in case of condensin (65), which forms a related protein complex to cohesin but which acts during condensation of mitotic or meiotic chromosomes. It is therefore possible that also cohesin has such an activity but it requires some specific conditions not found yet (66). However, it is also possible that cohesin is not a DNA/chromatin translocase and needs to be pushed along chromatin fibres by other motor proteins such as RNA polymerase (67) or by other mechanisms (68).

Recent experiments by Vian *et al*. (63) showed that cohesin that is unable to hydrolyse ATP, due to a mutation in ATPase motif, is still capable of diffusing along chromatin and reaching CTCF at TADs borders, although this occurs up to 2-fold less efficiently as compared to not-mutated cohesin. The authors interpreted this diminished efficiency as an indication that cohesin is a motor protein that requires ATP for its translocation along the chromatin, although they considered also the possibility that ATPase mutations decrease cohesin loading on chromatin fibres and this in turn decreases that amount of cohesin that reaches CTCF anchors (63). It seems though that the second possibility is more likely in light of studies showing that cohesin ATPase activity is required for cohesin loading on chromatin (69). Therefore, the experimental observation that cohesin that is unable to hydrolyse ATP and thus loads less efficiently still reaches its final destination in >50% of cases (63), suggests that it is not ATPase activity of cohesin that is implicated in its translocation but some other factors, such as transcription-induced supercoiling, for example.

*In vitro* experiments aimed to study diffusional motion of cohesin rings along DNA and chromatin established that cohesin rings embrace chromatin fibres very tightly, which results in a strong molecular friction between cohesin and enclosed chromatin fibre (70). That friction greatly limits spontaneous diffusion of cohesin rings along chromatin but does not block it completely (70). The same study concluded that it is unlikely that individual cohesin rings can embrace two chromatin fibres and therefore two cohesin rings forming so called cohesin handcuffs would be needed to span the growing loop during chromatin loop extrusion (70).

Another development important for the understanding of the possible involvement of supercoiling in chromatin loop extrusion consisted of establishing that type II DNA topoisomerases are bound to CTCF proteins forming borders of loop-forming TADs (71,72). This location of type II DNA topoisomerases suggests that torsional stress generated during transcription within individual TADs can be dissipated by diffusing towards TADs borders. Studies of Baranello *et al*. on the other hand explained better how the interplay between transcribing RNA polymerases and interacting with them type I DNA topoisomerases results in the preferential relaxation of positive supercoiling generated by transcribing RNA polymerase and thus in a net input of negative supercoiling by ongoing transcription (41).

Racko *et al*., incorporated the mentioned above elements into simulation studies testing whether transcription-induced supercoiling could drive chromatin loop extrusion (73). In the modelled system, handcuffs-forming cohesin rings were placed on chromatin fibres in such a way that a small chromatin loop formed between the two rings. It was assumed then that transcription starts in that small loop. This assumption agrees with studies showing that cohesin rings are preferentially loaded by Nibbl protein in the immediate vicinity of transcription start sites (TSS) of active genes and their enhancers (67,74). Instead of modelling RNA polymerase producing domains with positive and negative supercoiling and then modelling DNA topoisomerase relaxing preferentially positive supercoiling, Racko *et al*. in their simulations, introduced directly negative supercoiling into the chromatin portion between the two cohesin rings. In principle, when positive supercoils generated during transcription are preferentially relaxed by DNA topoisomerases, transcription is able to introduce one negative supercoil for every 10 nucleotides that are transcribed (75). This negative supercoiling is generated and initially confined to the chromatin portion behind transcribing RNA polymerase (24). Since transcribing RNA polymerase blocks the diffusion of supercoiling through the template portion it is actually bound to (24), dissociation of RNA polymerase would be needed to permit diffusion and equilibration of negative supercoiling in the entire chromatin loop between two cohesin rings. However, it is known that transcription cycles in well over 90% of cases undergo premature termination with RNA polymerase dissociating after synthesis of short 30–75 nt long RNAs (76,77). Racko *et al*., therefore assumed that RNA polymerase dissociation permits the negative supercoiling accumulated behind transcribing RNA polymerase to diffuse and equilibrate in the entire chromatin portion between two cohesin rings (73). It was also assumed that this process of injecting and equilibrating of negative supercoiling in the chromatin portion between two cohesin rings occurs repeatedly (73).

**Figure 4.**
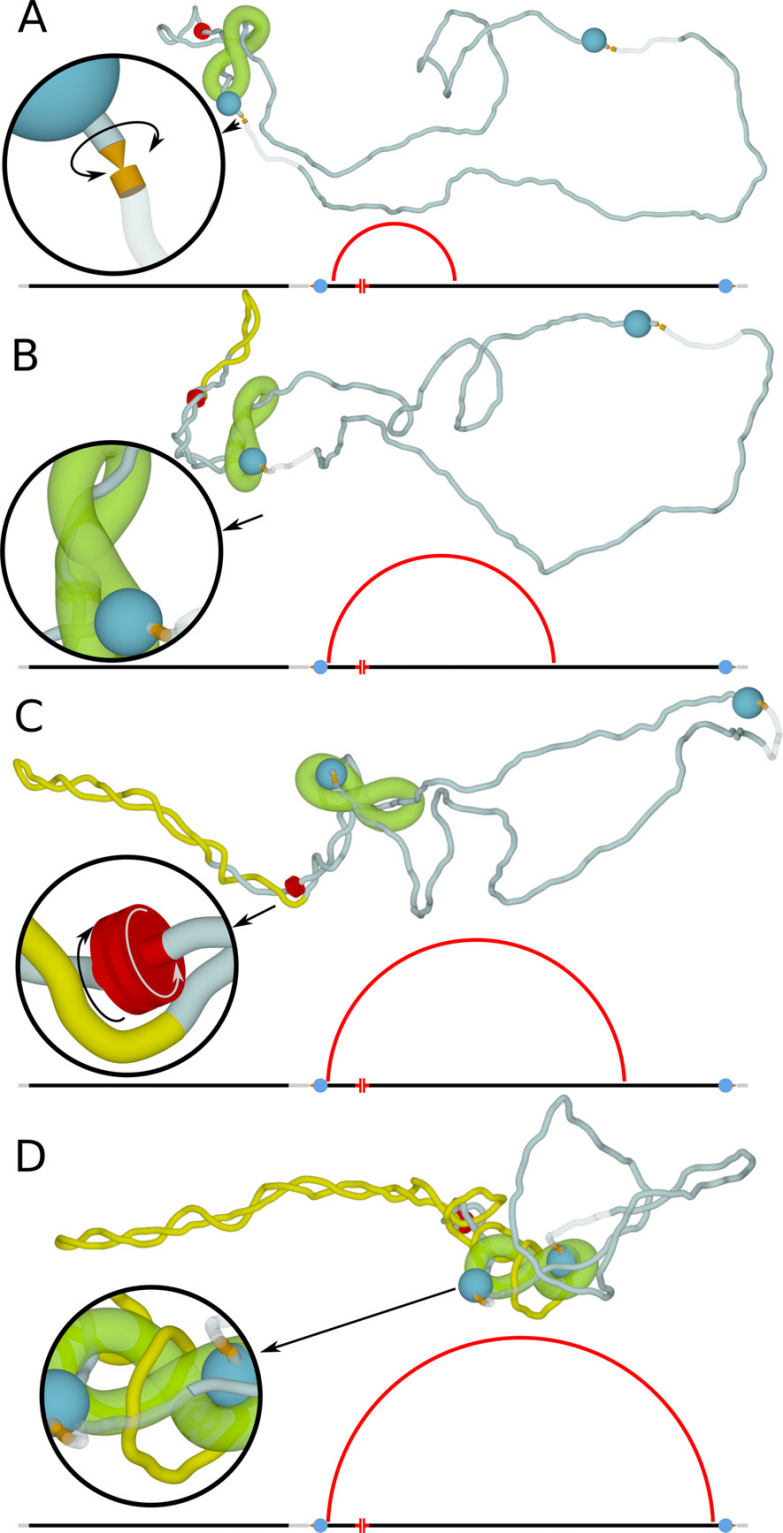
Transcription induced supercoiling drives chromatin loop extrusion and chromatin scanning by enhancers. (A-D) Snapshots from a simulation run where two cohesin rings forming a handcuff (modelled as rigid ‘8’, shown in green) are loaded on both sides of TSS of an enhancer (shown in red), which is positioned near one border of the simulated TAD. Transcription of eRNA is assumed here to be the main source of transcription-induced supercoiling responsible for TADs formation and therefore the enhancer here is modelled only as a motor introducing negative supercoiling (see inset in (C)). The border elements are regions with bound CTCF (modelled as spheres larger than the openings of the cohesin rings) and with bound Top2B, whose action in these regions is modelled as passive swivel sites permitting free axial rotation of chromatin fibres and as semi-transparent chain sections permitting other portions of modelled chromatin fibres to freely pass through (see inset in (A)). (**A**) Transcription-induced supercoiling starts to accumulate in the chromatin portion between two cohesin rings forming the handcuff. (**B**) Growing plectoneme pulls chromatin fibres through both cohesin rings forming the handcuff. However, pulling of chromatin fibres through one of the cohesin rings is stopped by CTCF protein bound at the border of the TAD (see inset for a magnified view). (**C, D**) As supercoiling is generated by ongoing transcription of eRNA, the plectoneme still grows by pulling one chromatin fibre through the unobstructed cohesin ring. The asymmetric loop extrusion enables the enhancer to progressively scan large stretches of chromatin fibre within the same TAD, which greatly facilitates the search for its cognate partner promoter. The scanned portion of chromatin fibre is shown as yellow in B, C and D. The loop extrusion stops when the second cohesin ring forming the handcuff is blocked by the CTCF bound to the second border of the TAD (see inset in (D)). The schematic diagrams below the snapshots present linear maps of the simulated TAD. The enhancer, shown in red, is placed near one of border elements composed of bound CTCF and sites of action of DNA topoisomerases. The red arcs present the progress of chromatin loop extrusion. Arcs join regions that in the corresponding snapshot are within the cohesin handcuffs. Figure 4 is adapted from (73).

As cohesin rings embrace tightly chromatin fibres and this slows down motion of chromatin fibres passing through cohesin rings (70) Racko *et al*. in their simulations increased hydrodynamic drag of regions of the chain that actually pass through modelled cohesin handcuffs. Accounting for this effect limits the dissipation of supercoiling via diffusion through cohesin rings forming the handcuff and thus favours accumulation of supercoiling in chromatin loops spanned by cohesin handcuffs (See Figure 4). Accounting for experimental demonstration that CTCF proteins defining boundaries of TADs are associated with type II DNA topoisomerases (71), Racko *et al*. introduced into modelled chromatin fibres short regions where segments can freely pass through each other and where they can also freely swivel (see Figure 4).

Figure 4 shows snapshots from simulations testing whether transcription-induced supercoiling can drive chromatin loop extrusion (73). Transcription occurring in the chromatin portion spanned by cohesin handcuffs causes formation of a plectoneme. As more supercoiling is introduced, the formed plectoneme grows and pushes the cohesin handcuffs towards the borders of the modelled TAD. That process can be also described as pulling of chromatin fibres through cohesin rings. The movement of chromatin fibres with respect to cohesin handcuffs is formally analogous to the process of chromatin loop extrusion proposed earlier (61,62). However, previous models assumed that cohesin has DNA/chromatin translocation activity or is pushed by some translocases, whereas simulations by Racko *et al*. showed that transcription-induced supercoiling is capable of driving chromatin loop extrusion (73).

**Figure 5.**
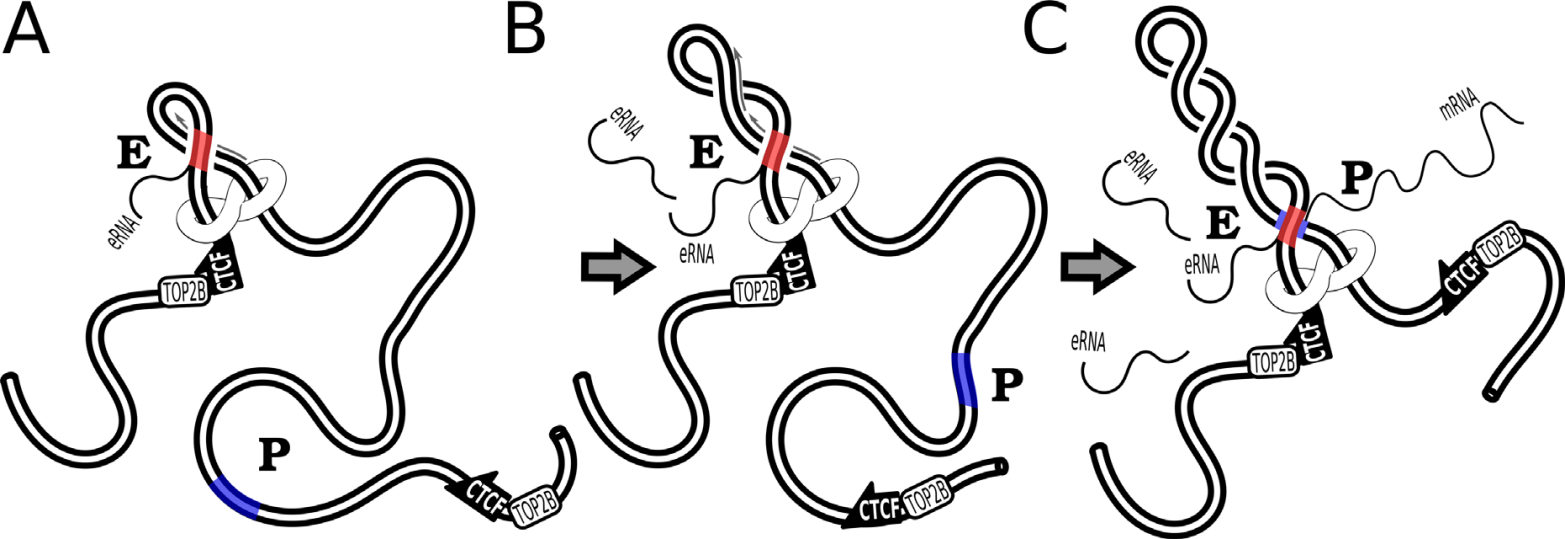
Schematic drawing explaining how an asymmetric loop extrusion starting from an enhancer located near CTCF sites drives chromatin scanning by that enhancer. (**A**) Cohesin rings preferentially load around transcribed enhancers (67,74). eRNA transcription, combined with the relaxation of positive supercoiling by Top1 (41,84), injects net negative supercoiling into the portion of chromatin fibre that is flanked by two cohesin rings. Due to the accumulated supercoiling, the enhancer region is drawn into a plectoneme and stays in a close contact with a stretch of chromatin that actually faces it. (**B, C**) While short eRNAs are produced, more negative supercoiling is injected causing the plectoneme to grow. Since one of cohesin rings is ‘clogged’ by its interaction with CTCF, the formed plectoneme can only grow and thus decrease its torsional stress by pulling chromatin fibre through the other cohesin ring forming the handcuff. The growth of plectoneme requires internal slithering motion that is well studied in case of supercoiled DNA (48). The portions of chromatin pulled through unclogged cohesin ring slide along the enhancer region. This sliding motion, or scanning, is the basis of a progressive 1D search during which enhancers with bound transcription factors can very efficiently find their cognate promoters to which they show affinity. Figure 5 is adapted from (73).

Very recent studies by Vian *et al*. (63) concluded that chromatin loop extrusion can serve as a reeling mechanism ensuring that active enhancers progressively scan chromatin regions separating them from their cognate promoters. For this to happen the two cohesin rings forming a handcuff should be placed initially on both sides of an enhancer TSS and then one of the rings should be immobilized by its stabilizing interaction with CTCF located close to the enhancer. Progressing chromatin loop extrusion permits then the enhancer to scan for stabilizing contacts within up to megabase large stretches of chromatin loops forming individual TADs. An analogous mechanism, but based on supercoiling-driven movement of chromatin fibres with respect of cohesin handcuffs, was proposed earlier (73) and is presented here in Figure 5.

It is important to stress here that the proposal involving supercoiling in formation of TADs critically depends on the presence of cohesin and is therefore entirely consistent with all studies showing the importance of cohesin in subdividing interphase chromosomes into TADs with CTCF-defined border elements (63,78–80).

There is growing evidence that transcription of enhancers and formation of enhancer promoter contacts precedes the transcription of genes activated by these enhancers (81,82) as it is also shown in the model in Figure 5. However, in the majority of cases the produced eRNAs does not seem to have a specific role and are quickly degraded (83). This has led to proposals that the mere act of transcription of enhancers is required for the activation of genes that may be located up to a megabase apart from their cognate enhancers (26,83). However, a mechanism linking the act of transcription of distal enhancers with formation of enhancer-promoter contact was missing. Simulation studies (73) showed that transcription induced supercoiling of eRNA is capable of driving chromatin loop extrusion that progressively slide enhancer region with respect of chromatin fibre forming a given TAD (see Figure 5). That mechanism can explain why many enhancers are transcribed and why the contacts between enhancers and their cognate promoters can be efficiently established even if their genomic distance is very large. However, enhancers and their cognate promoters need to be in the same TAD i.e. in a portion of chromatin that can be scanned by an enhancer before loop extrusion is stopped by interactions with border elements of TADs. To be able to generate the net input of negative supercoiling, which is needed to induce supercoiling flux, that in turn drives chromatin-loop extrusion, eRNA transcription should be similar to regular mRNA transcription with respect to preferential relaxation of positive supercoiling. Interestingly, studies of eRNA transcription showed that topoisomerase 1 colocalizes and relaxes DNA at sites of eRNA transcription (84), suggesting that at enhancers topoisomerase 1 might acts in a similar way as hypothesized for mRNA transcription (41).

If transcription-induced supercoiling drives chromatin loop extrusion, then blocking of transcription would be expected to abolish formation of TADs. Recent experiments by Vian *et al*. may suggest though that transcription is not needed for the formation of TADs as after inhibition of transcriptional elongation by flavopiridol the TADs were forming nearly normally in cultured mammalian cells (63). However, flavopiridol permits RNA polymerases to synthesize short RNA chains in the range of 30–75 nucleotides (76) and synthesis of these short chains can generate normal levels of transcription-induced supercoiling. Generation of supercoiling by abortive transcription may explain the biological sense of otherwise apparently wasteful process of abortive transcription where for no apparent biological gain well over 90% of RNA polymerases starting transcription dissociate after synthesizing only 30–75 nucleotide long RNA chains (77).

A significant confusion regarding the role of transcription in chromatin-loop extrusion may be caused by the fact that TADs in Drosophila were shown to form independently of transcription (85). However, TADs in Drosophila might not be formed by chromatin loop extrusion and/or that other mechanisms seem to play an important role (86). TADs in Drosophila are nanocompartments (87) i.e. short continuous chromatin portions with the same types of epigenetic marks. Direct or protein-mediated attraction between the same types of epigenetic marks causes then an increased frequency of internal contacts in these nanocompartments (87). The ‘hallmarks’ of the loop extrusion process, which are clearly visible in Hi-C contact maps of mammalian cells are focal peaks resulting from prolonged direct contacts between two borders of a given TAD (60,61). In mammalian cells, nearly half of TADs show such focal peaks (60) whereas in Drosophila cells such peaks are visible only in <1% of regions with increased frequency of contacts (86). As clearly stated by Eagen *et al*., ‘A close correlation between TADs and loops detectable by Hi-C is not conserved throughout metazoans.’ (86).

## CONCLUSIONS

We discussed here experimental data supporting the notion that TADs are supercoiled. We also presented how supercoiling of chromatin fibres can be generated during transcription and how supercoiling is expected to induce compaction of individual TADs and thus promote interaction between enhancers and their cognate promoters that are located in the same TAD. In addition, we propose that transcription-induced supercoiling is likely to provide a long-sought solution to the question of how chromatin loop extrusion is driven and explains the mechanical role of eRNA transcription. New experimental approaches, such as single cell Hi-C (88) are needed, however, to reveal to us the structure of individual TADs. It is important to add here that transcription-induced supercoiling would be only needed to stimulate the expression of genes positively regulated by contacts with distal regulatory elements. Transcription of eRNA or of house-keeping genes which do not need an activation by interaction with distal regulatory elements can be initiated in the absence of supercoiling and can be used then as the source of supercoiling activating other genes.

## References

[1] Friedmann H.C. From “butyribacterium” to “E. coli”: an essay on unity in biochemistry. Perspect. Biol. Med. 2004; 47:47–66. 15061168 10.1353/pbm.2004.0007

[2] Stasiak A., Egelman E.H. Structure and function of RecA-DNA complexes. Cell. Mol. Life Sci. 1994; 50:192–203. 10.1007/BF01924002 8143793

[3] Kleinschmidt A., Lang D., Zahn R.K. Uber die intrazellulare formation von Bacterien - DNS. Z. Naturforschg. 1961; 16 b:730–739.

[4] Kavenoff R., Bowen B.C. Electron microscopy of membrane-free folded chromosomes from Escherichia coli. Chromosoma. 1976; 59:89–101. 795620 10.1007/BF00328479

[5] Postow L., Hardy C.D., Arsuaga J., Cozzarelli N.R. Topological domain structure of the Escherichia coli chromosome. Genes Dev. 2004; 18:1766–1779. 15256503 10.1101/gad.1207504 PMC478196

[6] Travers A., Muskhelishvili G. DNA supercoiling - a global transcriptional regulator for enterobacterial growth. Nat. Rev. Microbiol. 2005; 3:157–169. 15685225 10.1038/nrmicro1088

[7] Paulson J.R., Laemmli U.K. The structure of histone-depleted metaphase chromosomes. Cell. 1977; 12:817–828. 922894 10.1016/0092-8674(77)90280-x

[8] Mirkovitch J., Mirault M.E., Laemmli U.K. Organization of the higher-order chromatin loop: specific DNA attachment sites on nuclear scaffold. Cell. 1984; 39:223–232. 6091913 10.1016/0092-8674(84)90208-3

[9] Luderus M.E., de Graaf A., Mattia E., den Blaauwen J.L., Grande M.A., de Jong L., van Driel R. Binding of matrix attachment regions to lamin B1. Cell. 1992; 70:949–959. 1525831 10.1016/0092-8674(92)90245-8

[10] Sperry A.O., Blasquez V.C., Garrard W.T. Dysfunction of chromosomal loop attachment sites: illegitimate recombination linked to matrix association regions and topoisomerase II. Proc. Natl. Acad. Sci. U.S.A. 1989; 86:5497–5501. 2546156 10.1073/pnas.86.14.5497 PMC297650

[11] Nelson W.G., Pienta K.J., Barrack E.R., Coffey D.S. The role of the nuclear matrix in the organization and function of DNA. Annu. Rev. Biophys. Biophys. Chem. 1986; 15:457–475. 3013231 10.1146/annurev.bb.15.060186.002325

[12] Jackson D.A., Dickinson P., Cook P.R. The size of chromatin loops in HeLa cells. EMBO J. 1990; 9:567–571. 2303042 10.1002/j.1460-2075.1990.tb08144.x PMC551702

[13] McDowall A.W., Smith J.M., Dubochet J. Cryo-electron microscopy of vitrified chromosomes in situ. EMBO J. 1986; 5:1395–1402. 3755397 10.1002/j.1460-2075.1986.tb04373.x PMC1166954

[14] Grosberg A., Rabin Y., Havlin S., Neer A. Crumpled globule model of the three-dimensional structure of DNA. Europhys. Lett. 1993; 23:373.

[15] Rosa A., Everaers R. Structure and dynamics of interphase chromosomes. PLoS Comput. Biol. 2008; 4:e1000153. 18725929 10.1371/journal.pcbi.1000153 PMC2515109

[16] Lieberman-Aiden E., van Berkum N.L., Williams L., Imakaev M., Ragoczy T., Telling A., Amit I., Lajoie B.R., Sabo P.J., Dorschner M.O. Comprehensive mapping of long-range interactions reveals folding principles of the human genome. Science. 2009; 326:289–293. 19815776 10.1126/science.1181369 PMC2858594

[17] Dixon J.R., Selvaraj S., Yue F., Kim A., Li Y., Shen Y., Hu M., Liu J.S., Ren B. Topological domains in mammalian genomes identified by analysis of chromatin interactions. Nature. 2012; 485:376–380. 22495300 10.1038/nature11082 PMC3356448

[18] Nora E.P., Lajoie B.R., Schulz E.G., Giorgetti L., Okamoto I., Servant N., Piolot T., van Berkum N.L., Meisig J., Sedat J. Spatial partitioning of the regulatory landscape of the X-inactivation centre. Nature. 2012; 485:381–385. 22495304 10.1038/nature11049 PMC3555144

[19] Sexton T., Yaffe E., Kenigsberg E., Bantignies F., Leblanc B., Hoichman M., Parrinello H., Tanay A., Cavalli G. Three-dimensional folding and functional organization principles of the Drosophila genome. Cell. 2012; 148:458–472. 22265598 10.1016/j.cell.2012.01.010

[20] Barbieri M., Chotalia M., Fraser J., Lavitas L.M., Dostie J., Pombo A., Nicodemi M. Complexity of chromatin folding is captured by the strings and binders switch model. Proc. Natl. Acad. Sci. U.S.A. 2012; 109:16173–16178. 22988072 10.1073/pnas.1204799109 PMC3479593

[21] Benedetti F., Dorier J., Burnier Y., Stasiak A. Models that include supercoiling of topological domains reproduce several known features of interphase chromosomes. Nucleic Acids Res. 2014; 42:2848–2855. 24366878 10.1093/nar/gkt1353 PMC3950722

[22] Liu L.F., Wang J.C. Supercoiling of the DNA template during transcription. Proc. Natl. Acad. Sci. U.S.A. 1987; 84:7024–7027. 2823250 10.1073/pnas.84.20.7024 PMC299221

[23] Wu H.Y., Shyy S.H., Wang J.C., Liu L.F. Transcription generates positively and negatively supercoiled domains in the template. Cell. 1988; 53:433–440. 2835168 10.1016/0092-8674(88)90163-8

[24] Ma J., Bai L., Wang M.D. Transcription under torsion. Science. 2013; 340:1580–1583. 23812716 10.1126/science.1235441 PMC5657242

[25] Ma J., Wang M.D. DNA supercoiling during transcription. Biophys. Rev. 2016; 8:75–87. 10.1007/s12551-016-0215-9 PMC5338639 28275417

[26] Kouzine F., Levens D., Baranello L. DNA topology and transcription. Nucleus. 2014; 5:195–202. 24755522 10.4161/nucl.28909 PMC4133214

[27] Bjorkegren C., Baranello L. DNA Supercoiling, topoisomerases, and Cohesin: Partners in regulating chromatin architecture. Int. J. Mol. Sci. 2018; 19:E884. 29547555 10.3390/ijms19030884 PMC5877745

[28] Papantonis A., Cook P.R. Transcription factories: genome organization and gene regulation. Chem. Rev. 2013; 113:8683–8705. 23597155 10.1021/cr300513p

[29] Nelson P. Transport of torsional stress in DNA. Proc. Natl. Acad. Sci. U.S.A. 1999; 96:14342–14347. 10588707 10.1073/pnas.96.25.14342 PMC24438

[30] Koster D.A., Croquette V., Dekker C., Shuman S., Dekker N.H. Friction and torque govern the relaxation of DNA supercoils by eukaryotic topoisomerase IB. Nature. 2005; 434:671–674. 15800630 10.1038/nature03395

[31] Bermudez I., Garcia-Martinez J., Perez-Ortin J.E., Roca J. A method for genome-wide analysis of DNA helical tension by means of psoralen-DNA photobinding. Nucleic Acids Res. 2010; 38:e182. 20685815 10.1093/nar/gkq687 PMC2965259

[32] Fernandez X., Diaz-Ingelmo O., Martinez-Garcia B., Roca J. Chromatin regulates DNA torsional energy via topoisomerase II-mediated relaxation of positive supercoils. EMBO J. 2014; 33:1492–1501. 24859967 10.15252/embj.201488091 PMC4194091

[33] Ljungman M., Hanawalt P.C. Localized torsional tension in the DNA of human cells. Proc. Natl. Acad. Sci. U.S.A. 1992; 89:6055–6059. 1631091 10.1073/pnas.89.13.6055 PMC49436

[34] Ljungman M., Hanawalt P.C. Presence of negative torsional tension in the promoter region of the transcriptionally poised dihydrofolate reductase gene in vivo. Nucleic Acids Res. 1995; 23:1782–1789. 7784183 10.1093/nar/23.10.1782 PMC306936

[35] Kramer P.R., Sinden R.R. Measurement of unrestrained negative supercoiling and topological domain size in living human cells. Biochemistry. 1997; 36:3151–3158. 9115991 10.1021/bi962396q

[36] Naughton C., Avlonitis N., Corless S., Prendergast J.G., Mati I.K., Eijk P.P., Cockroft S.L., Bradley M., Ylstra B., Gilbert N. Transcription forms and remodels supercoiling domains unfolding large-scale chromatin structures. Nat. Struct. Mol. Biol. 2013; 20:387–395. 23416946 10.1038/nsmb.2509 PMC3689368

[37] Kouzine F., Gupta A., Baranello L., Wojtowicz D., Ben-Aissa K., Liu J., Przytycka T.M., Levens D. Transcription-dependent dynamic supercoiling is a short-range genomic force. Nat. Struct. Mol. Biol. 2013; 20:396–403. 23416947 10.1038/nsmb.2517 PMC3594045

[38] Teves S.S., Henikoff S. Transcription-generated torsional stress destabilizes nucleosomes. Nat. Struct. Mol. Biol. 2014; 21:88–94. 24317489 10.1038/nsmb.2723 PMC3947361

[39] Bates A.D., Maxwell A. DNA Topology. 2005; Oxford: Oxford University Press.

[40] Phillips-Cremins J.E., Sauria M.E., Sanyal A., Gerasimova T.I., Lajoie B.R., Bell J.S., Ong C.T., Hookway T.A., Guo C., Sun Y. Architectural protein subclasses shape 3D organization of genomes during lineage commitment. Cell. 2013; 153:1281–1295. 23706625 10.1016/j.cell.2013.04.053 PMC3712340

[41] Baranello L., Wojtowicz D., Cui K., Devaiah B.N., Chung H.J., Chan-Salis K.Y., Guha R., Wilson K., Zhang X., Zhang H. RNA polymerase II regulates topoisomerase 1 activity to favor efficient transcription. Cell. 2016; 165:357–371. 27058666 10.1016/j.cell.2016.02.036 PMC4826470

[42] Smith G.R. DNA supercoiling: another level for regulating gene expression. Cell. 1981; 24:599–600. 6265097 10.1016/0092-8674(81)90085-4

[43] Schultz M.C., Brill S.J., Ju Q., Sternglanz R., Reeder R.H. Topoisomerases and yeast rRNA transcription: negative supercoiling stimulates initiation and topoisomerase activity is required for elongation. Genes Dev. 1992; 6:1332–1341. 1321070 10.1101/gad.6.7.1332

[44] Dunaway M., Ostrander E.A. Local domains of supercoiling activate a eukaryotic promoter in vivo. Nature. 1993; 361:746–748. 8441472 10.1038/361746a0

[45] Brackley C.A., Johnson J., Bentivoglio A., Corless S., Gilbert N., Gonnella G., Marenduzzo D. Stochastic Model of Supercoiling-Dependent Transcription. Phys. Rev. Lett. 2016; 117:018101. 27419594 10.1103/PhysRevLett.117.018101

[46] Lupianez D.G., Kraft K., Heinrich V., Krawitz P., Brancati F., Klopocki E., Horn D., Kayserili H., Opitz J.M., Laxova R. Disruptions of topological chromatin domains cause pathogenic rewiring of gene-enhancer interactions. Cell. 2015; 161:1012–1025. 25959774 10.1016/j.cell.2015.04.004 PMC4791538

[47] Vologodskii A.V., Levene S.D., Klenin K.V., Frank-Kamenetskii M., Cozzarelli N.R. Conformational and thermodynamic properties of supercoiled DNA. J. Mol. Biol. 1992; 227:1224–1243. 1433295 10.1016/0022-2836(92)90533-p

[48] Huang J., Schlick T., Vologodskii A. Dynamics of site juxtaposition in supercoiled DNA. Proc. Natl. Acad. Sci. U.S.A. 2001; 98:968–973. 11158579 10.1073/pnas.98.3.968 PMC14693

[49] Bednar J., Furrer P., Stasiak A., Dubochet J., Egelman E.H., Bates A.D. The twist, writhe and overall shape of supercoiled DNA change during counterion-induced transition from a loosely to a tightly interwound superhelix. Possible implications for DNA structure in vivo. J. Mol. Biol. 1994; 235:825–847. 8289322 10.1006/jmbi.1994.1042

[50] Weintraub A.S., Li C.H., Zamudio A.V., Sigova A.A., Hannett N.M., Day D.S., Abraham B.J., Cohen M.A., Nabet B., Buckley D.L. YY1 is a structural regulator of Enhancer-Promoter loops. Cell. 2017; 171:1573–1588. 29224777 10.1016/j.cell.2017.11.008 PMC5785279

[51] Benedetti F., Dorier J., Stasiak A. Effects of supercoiling on enhancer-promoter contacts. Nucleic Acids Res. 2014; 42:10425–10432. 25123662 10.1093/nar/gku759 PMC4176356

[52] Zabidi M.A., Arnold C.D., Schernhuber K., Pagani M., Rath M., Frank O., Stark A. Enhancer-core-promoter specificity separates developmental and housekeeping gene regulation. Nature. 2015; 518:556–559. 25517091 10.1038/nature13994 PMC6795551

[53] Hu Z., Tee W.W. Enhancers and chromatin structures: regulatory hubs in gene expression and diseases. Biosci. Rep. 2017; 37:doi:10.1042/BSR20160183. 10.1042/BSR20160183 PMC5408663 28351896

[54] Jin F., Li Y., Dixon J.R., Selvaraj S., Ye Z., Lee A.Y., Yen C.A., Schmitt A.D., Espinoza C.A., Ren B. A high-resolution map of the three-dimensional chromatin interactome in human cells. Nature. 2013; 503:290–294. 24141950 10.1038/nature12644 PMC3838900

[55] Heinz S., Benner C., Spann N., Bertolino E., Lin Y.C., Laslo P., Cheng J.X., Murre C., Singh H., Glass C.K. Simple combinations of lineage-determining transcription factors prime cis-regulatory elements required for macrophage and B cell identities. Mol. Cell. 2010; 38:576–589. 20513432 10.1016/j.molcel.2010.05.004 PMC2898526

[56] Ferrandiz M.J., Martin-Galiano A.J., Schvartzman J.B., de la Campa A.G. The genome of Streptococcus pneumoniae is organized in topology-reacting gene clusters. Nucleic Acids Res. 2010; 38:3570–3581. 20176571 10.1093/nar/gkq106 PMC2887967

[57] Dorman C.J., Dorman M.J. DNA supercoiling is a fundamental regulatory principle in the control of bacterial gene expression. Biophys. Rev. 2016; 8:89–100. 28510216 10.1007/s12551-016-0238-2 PMC5418507

[58] Meyer S., Reverchon S., Nasser W., Muskhelishvili G. Chromosomal organization of transcription: in a nutshell. Curr. Genet. 2018; 64:555–565. 29184972 10.1007/s00294-017-0785-5

[59] Chong S., Chen C., Ge H., Xie X.S. Mechanism of transcriptional bursting in bacteria. Cell. 2014; 158:314–326. 25036631 10.1016/j.cell.2014.05.038 PMC4105854

[60] Rao S.S., Huntley M.H., Durand N.C., Stamenova E.K., Bochkov I.D., Robinson J.T., Sanborn A.L., Machol I., Omer A.D., Lander E.S. A 3D map of the human genome at kilobase resolution reveals principles of chromatin looping. Cell. 2014; 159:1665–1680. 25497547 10.1016/j.cell.2014.11.021 PMC5635824

[61] Sanborn A.L., Rao S.S., Huang S.C., Durand N.C., Huntley M.H., Jewett A.I., Bochkov I.D., Chinnappan D., Cutkosky A., Li J. Chromatin extrusion explains key features of loop and domain formation in wild-type and engineered genomes. Proc. Natl. Acad. Sci. U.S.A. 2015; 112:E6456–E6465. 26499245 10.1073/pnas.1518552112 PMC4664323

[62] Fudenberg G., Imakaev M., Lu C., Goloborodko A., Abdennur N., Mirny L.A. Formation of chromosomal domains by loop extrusion. Cell Rep. 2016; 15:2038–2049. 27210764 10.1016/j.celrep.2016.04.085 PMC4889513

[63] Vian L., Pekowska A., Rao S.S.P., Kieffer-Kwon K.R., Jung S., Baranello L., Huang S.C., El Khattabi L., Dose M., Pruett N. The energetics and physiological impact of cohesin extrusion. Cell. 2018; 173:1165–1178. 29706548 10.1016/j.cell.2018.03.072 PMC6065110

[64] Xiao T., Wallace J., Felsenfeld G. Specific sites in the C terminus of CTCF interact with the SA2 subunit of the cohesin complex and are required for cohesin-dependent insulation activity. Mol. Cell. Biol. 2011; 31:2174–2183. 21444719 10.1128/MCB.05093-11 PMC3133248

[65] Ganji M., Shaltiel I.A., Bisht S., Kim E., Kalichava A., Haering C.H., Dekker C. Real-time imaging of DNA loop extrusion by condensin. Science. 2018; 360:102–105. 29472443 10.1126/science.aar7831 PMC6329450

[66] Fudenberg G., Abdennur N., Imakaev M., Goloborodko A., Mirny L.A. Emerging evidence of chromosome folding by loop extrusion. Cold Spring Harb. Symp. Quant. Biol. 2017; 82:45–55. 29728444 10.1101/sqb.2017.82.034710 PMC6512960

[67] Busslinger G.A., Stocsits R.R., van der Lelij P., Axelsson E., Tedeschi A., Galjart N., Peters J.M. Cohesin is positioned in mammalian genomes by transcription, CTCF and Wapl. Nature. 2017; 544:503–507. 28424523 10.1038/nature22063 PMC6080695

[68] Brackley C.A., Johnson J., Michieletto D., Morozov A.N., Nicodemi M., Cook P.R., Marenduzzo D. Extrusion without a motor: a new take on the loop extrusion model of genome organization. Nucleus. 2018; 9:95–103. 29300120 10.1080/19491034.2017.1421825 PMC5973195

[69] Ladurner R., Bhaskara V., Huis in ’t Veld P.J., Davidson I.F., Kreidl E., Petzold G., Peters J.M. Cohesin's ATPase activity couples cohesin loading onto DNA with Smc3 acetylation. Curr. Biol.: CB. 2014; 24:2228–2237. 25220052 10.1016/j.cub.2014.08.011 PMC4188815

[70] Stigler J., Camdere G.O., Koshland D.E., Greene E.C. Single-Molecule imaging reveals a collapsed conformational state for DNA-Bound cohesin. Cell Rep. 2016; 15:988–998. 27117417 10.1016/j.celrep.2016.04.003 PMC4856582

[71] Uuskula-Reimand L., Hou H., Samavarchi-Tehrani P., Rudan M.V., Liang M., Medina-Rivera A., Mohammed H., Schmidt D., Schwalie P., Young E.J. Topoisomerase II beta interacts with cohesin and CTCF at topological domain borders. Genome Biol. 2016; 17:182. 27582050 10.1186/s13059-016-1043-8 PMC5006368

[72] Canela A., Maman Y., Jung S., Wong N., Callen E., Day A., Kieffer-Kwon K.R., Pekowska A., Zhang H., Rao S.S.P. Genome organization drives chromosome fragility. Cell. 2017; 170:507–521. 28735753 10.1016/j.cell.2017.06.034 PMC6133249

[73] Racko D., Benedetti F., Dorier J., Stasiak A. Transcription-induced supercoiling as the driving force of chromatin loop extrusion during formation of TADs in interphase chromosomes. Nucleic Acids Res. 2018; 46:1648–1660. 29140466 10.1093/nar/gkx1123 PMC5829651

[74] Kagey M.H., Newman J.J., Bilodeau S., Zhan Y., Orlando D.A., van Berkum N.L., Ebmeier C.C., Goossens J., Rahl P.B., Levine S.S. Mediator and cohesin connect gene expression and chromatin architecture. Nature. 2010; 467:430–435. 20720539 10.1038/nature09380 PMC2953795

[75] Gilbert N., Allan J. Supercoiling in DNA and chromatin. Curr. Opin. Genet. Dev. 2014; 25:15–21. 24584092 10.1016/j.gde.2013.10.013 PMC4042020

[76] Nilson K.A., Lawson C.K., Mullen N.J., Ball C.B., Spector B.M., Meier J.L., Price D.H. Oxidative stress rapidly stabilizes promoter-proximal paused Pol II across the human genome. Nucleic Acids Res. 2017; 45:11088–11105. 28977633 10.1093/nar/gkx724 PMC5737879

[77] Steurer B., Janssens R.C., Geverts B., Geijer M.E., Wienholz F., Theil A.F., Chang J., Dealy S., Pothof J., van Cappellen W.A. Live-cell analysis of endogenous GFP-RPB1 uncovers rapid turnover of initiating and promoter-paused RNA Polymerase II. Proc. Natl. Acad. Sci. U.S.A. 2018; 115:E4368–E4376. 29632207 10.1073/pnas.1717920115 PMC5948963

[78] Wutz G., Varnai C., Nagasaka K., Cisneros D.A., Stocsits R.R., Tang W., Schoenfelder S., Jessberger G., Muhar M., Hossain M.J. Topologically associating domains and chromatin loops depend on cohesin and are regulated by CTCF, WAPL, and PDS5 proteins. EMBO J. 2017; 36:3573–3599. 29217591 10.15252/embj.201798004 PMC5730888

[79] Schwarzer W., Abdennur N., Goloborodko A., Pekowska A., Fudenberg G., Loe-Mie Y., Fonseca N.A., Huber W., C H.H., Mirny L. Two independent modes of chromatin organization revealed by cohesin removal. Nature. 2017; 551:51–56. 29094699 10.1038/nature24281 PMC5687303

[80] Rao S.S.P., Huang S.C., Glenn St Hilaire B., Engreitz J.M., Perez E.M., Kieffer-Kwon K.R., Sanborn A.L., Johnstone S.E., Bascom G.D., Bochkov I.D. Cohesin loss eliminates all loop domains. Cell. 2017; 171:305–320. 28985562 10.1016/j.cell.2017.09.026 PMC5846482

[81] Arner E., Daub C.O., Vitting-Seerup K., Andersson R., Lilje B., Drablos F., Lennartsson A., Ronnerblad M., Hrydziuszko O., Vitezic M. Transcribed enhancers lead waves of coordinated transcription in transitioning mammalian cells. Science. 2015; 347:1010–1014. 25678556 10.1126/science.1259418 PMC4681433

[82] Kim Y.W., Lee S., Yun J., Kim A. Chromatin looping and eRNA transcription precede the transcriptional activation of gene in the beta-globin locus. Biosci. Rep. 2015; 35:e00179. 25588787 10.1042/BSR20140126 PMC4370096

[83] Li W., Notani D., Rosenfeld M.G. Enhancers as non-coding RNA transcription units: recent insights and future perspectives. Nat. Rev. Genet. 2016; 17:207–223. 26948815 10.1038/nrg.2016.4

[84] Puc J., Kozbial P., Li W., Tan Y., Liu Z., Suter T., Ohgi K.A., Zhang J., Aggarwal A.K., Rosenfeld M.G. Ligand-dependent enhancer activation regulated by topoisomerase-I activity. Cell. 2015; 160:367–380. 25619691 10.1016/j.cell.2014.12.023 PMC4422651

[85] Hug C.B., Grimaldi A.G., Kruse K., Vaquerizas J.M. Chromatin architecture emerges during zygotic genome activation independent of transcription. Cell. 2017; 169:216–228. 28388407 10.1016/j.cell.2017.03.024

[86] Eagen K.P., Aiden E.L., Kornberg R.D. Polycomb-mediated chromatin loops revealed by a subkilobase-resolution chromatin interaction map. Proc. Natl. Acad. Sci. U.S.A. 2017; 114:8764–8769. 28765367 10.1073/pnas.1701291114 PMC5565414

[87] Szabo Q., Jost D., Chang J.M., Cattoni D.I., Papadopoulos G.L., Bonev B., Sexton T., Gurgo J., Jacquier C., Nollmann M. TADs are 3D structural units of higher-order chromosome organization in Drosophila. Sci. Adv. 2018; 4:eaar8082. 29503869 10.1126/sciadv.aar8082 PMC5829972

[88] Stevens T.J., Lando D., Basu S., Atkinson L.P., Cao Y., Lee S.F., Leeb M., Wohlfahrt K.J., Boucher W., O'Shaughnessy-Kirwan A. 3D structures of individual mammalian genomes studied by single-cell Hi-C. Nature. 2017; 544:59–64. 28289288 10.1038/nature21429 PMC5385134

[89] Jen-Jacobson L. Protein-DNA recognition complexes: conservation of structure and binding energy in the transition state. Biopolymers. 1997; 44:153–180. 9354759 10.1002/(SICI)1097-0282(1997)44:2<153::AID-BIP4>3.0.CO;2-U

